# Rapid and highly sensitive approach for multiplexed somatic fusion detection

**DOI:** 10.1038/s41379-022-01058-y

**Published:** 2022-03-28

**Authors:** Samuel Abbou, Sarah Finstuen-Magro, Brigit McDannell, Michelle Feenstra, Abigail Ward, David S. Shulman, Birgit Geoerger, Joadly Duplan, Hannah Comeau, Katherine A. Janeway, Kelly Klega, Brian D. Crompton

**Affiliations:** 1 Dana-Farber/Boston Children’s Cancer and Blood Disorders Center, Boston, MA USA; 2 Gustave Roussy Cancer Campus, Department of Pediatric and Adolescent Oncology, INSERM U1015, Université Paris-Saclay, Villejuif, France; 3 Broad Institute of Harvard and MIT, Cambridge, MA USA

**Keywords:** Sarcoma, Cancer genetics

## Abstract

Somatic gene translocations are key to making an accurate diagnosis in many cancers including many pediatric sarcomas. Currently available molecular diagnostic approaches to identifying somatic pathognomonic translocations have limitations such as minimal multiplexing, high cost, complex computational requirements, or slow turnaround times. We sought to develop a new fusion-detection assay optimized to mitigate these challenges. To accomplish this goal, we developed a highly sensitive multiplexed digital PCR-based approach that can identify the gene partners of multiple somatic fusion transcripts. This assay was validated for specificity with cell lines and synthetized DNA fragments. Assay sensitivity was optimized using a tiered amplification approach for fusion detection from low input and/or degraded RNA. The assay was then tested for the potential application of fusion detection from FFPE tissue and liquid biopsy samples. We found that this multiplexed PCR approach was able to accurately identify the presence of seven different targeted fusion transcripts with a turnaround time of 1 to 2 days. The addition of a tiered amplification step allowed the detection of targeted fusions from as little as 1 pg of RNA input. We also identified fusions from as little as two unstained slides of FFPE tumor biopsy tissue, from circulating tumor cells collected from tumor-bearing mice, and from liquid biopsy samples from patients with known fusion-positive cancers. We also demonstrated that the assay could be easily adapted for additional fusion targets. In summary, this novel assay detects multiple somatic fusion partners in biologic samples with low tumor content and low-quality RNA in less than two days. The assay is inexpensive and could be applied to surgical and liquid biopsies, particularly in places with inadequate resources for more expensive and expertise-dependent assays such as next-generation sequencing.

## Introduction

Somatic gene translocations that give rise to oncogenic fusion transcripts characterize many cancers, especially many types of pediatric solid tumor malignancies. Illustrative examples include Ewing sarcoma (EWS), defined by fusions of the *EWSR1* gene with one of the *ETS* transcription factor genes and fusion-positive rhabdomyosarcoma (RMS), expressing PAX3- or PAX7-FOXO1 (PAX3/7-FOXO1) fusion transcripts^1,2^. Identification of these fusions have become increasingly important for making an accurate pathologic diagnosis, especially now that we recognize entities that are histologically similar to common fusion-driven cancers but harbor different genomics and respond differently to treatment. These include small round blue cell tumors that share some pathologic characteristics of EWS but express alternative fusions, such as BCOR-CCNB3 and CIC-DUX4, and appear to have different expected outcomes when treated with Ewing sarcoma specific therapy^3,4^. Similarly, alveolar RMS tumors, which are most commonly characterized by PAX3/7-FOXO1 fusions, are sometimes found to be fusion negative and respond to therapy similarly to fusion-negative embryonal rhabdomyosarcoma^5^. In fact, the most recent World Health Organization Classification of Tumors of Soft Tissue and Bone now defines distinct genetic subsets of pediatric sarcomas by the presence or absence of specific fusion partners^6^. Molecular identification of these fusions is now commonly felt to be required for accurate diagnosis of many of these malignancies.

Current approaches to identify chromosomal rearrangements or their resultant fusion transcripts include fluorescent in-situ hybridization (FISH), DNA-sequencing, RNA-sequencing, reverse transcriptase polymerase chain reaction (RT-PCR), and combinations of PCR and sequencing. Limitations of these technologies include a minimal ability to test for multiple fusions simultaneously, as in FISH, long reporting times and high cost, as in sequencing-based studies, and requirements of most of these assays for relatively large quantities of high-quality tissue material. In some cases, it is challenging to meet these requirements or to await the needed time to obtain results.

In this study, we set out to design a highly sensitive and highly specific assay that could accurately identify genetic partners of a somatic fusion transcript from minimal tumor biopsy material or from a liquid biopsy sample in a patient with an undiagnosed tumor that could be performed and interpreted in less than 48 h. To do this, we developed a multiplexed droplet digital PCR (ddPCR) strategy that satisfies these criteria and is flexible enough to be quickly adapted to detect additional translocations of interest. We validated this approach in samples collected from patients with EWS, fusion-positive RMS, desmoplastic small round cell tumors (DSRCT) and small round blue cell tumors with non-EWS fusions, including the BCOR-CCNB3 fusion. We demonstrate that it is feasible to identify multiple disease-defining translocations from extremely limited tumor biopsy and liquid biopsy material with a single low-cost assay with a short time to reporting of results.

## Materials and methods

### Cell lines and synthetic DNA construct

As positive controls, cDNA from cell lines A673, TTC466, RH4 and CW9019 were used because they express the EWSR1-FLI1, EWSR1-ERG, PAX3-FOXO1 and PAX7-FOXO1 fusion transcripts respectively. The cell line 293T was used as fusion negative control line. Positive and negative controls were used in every experiment. The cells were grown in RPMI 1640 (TTC466, RH4, CW9019, 293T) or DMEM (A673) media with 2 mM L-glutamine, and 10% heat-inactivated fetal calf serum. Synthetic DNA was used for the BCOR-CCNB3, EWSR1-WT1 and FUS-ERG fusions (Integrated DNA Technologies, IA, USA) since cell lines with these translocations were not available.

### In Vivo mouse experiment

NOD scid gamma (NSG) 6-weeks old female mice were used. Mice were injected with 5 × 10^5^ luciferase labeled Ewing sarcoma cells, including either A673 or TC71, through the tail vein. Mouse health was monitored with weekly body weight measurements. Tumor burden was monitored weekly using the IVIS Spectrum In Vivo Imaging System (PerkinElmer, MA, USA). To do this, mice were injected subcutaneously with 75 mg/kg D-luciferin potassium salt (Promega E1605) in sterile PBS and anesthetized with 2% isoflurane in medical air. Serial bioluminescence images were acquired using the automated exposure set-up. The peak bioluminescence signal intensity within selected regions of interest (ROI) was quantified using the Living Image Software (PerkinElmer) and expressed as photon flux (p/sec/cm2/sr). Relative bioluminescence is calculated by a ratio of bioluminescence at day 28 and day 7, BLI_day28/_BLI_day7_.

Mice were sacrificed at day 28, or when the mice showed disease symptoms, and terminal blood was collected by cardiac puncture. All blood samples were transferred on ice to the laboratory right after collection. Blood was collected in EDTA tubes and kept at 4 °C until processed. ScreenCell MB kit (ScreenCell, Sarcelles, France) was used with an adapted protocol. Mouse blood was diluted in at least 2 mL of PBS before a gradient separation step. Samples were transferred to the ScreenCell device after dilution to a total volume of 7 mL with PBS. The sample was then processed as described below.

### Patients and samples processing for circulating tumor cell (CTC) enrichment

Patients at the Dana-Farber Cancer Institute and Boston Children’s Hospital were consented to one or more IRB approved protocols. Blood and bone marrow samples were collected in K2EDTA or Cell-Free DNA BCT tubes (Streck, Nebraska, USA). Effusion samples were collected in a sterile container and 10 mL were used. All samples were centrifuged for 10 min at 1000–1200 g. Plasma or supernatant was transferred to a new tube and centrifuged for 10 min at 2500 × *g* and stored for other purposes. Cell layer from the first centrifugation and pellet from the second were pooled together for CTC enrichment. DNA from patient samples without any translocation specific diseases were used as negative controls.

Celsee PREP 100 with Celsee CTC enrichment Kit (Bio-Rad laboratories, CA, USA) or ScreenCell MB kit were used for blood or bone marrow CTC enrichment with an adapted protocol. For enrichment with Celsee PREP 100, the sample was diluted 1:1, after plasma was removed, with a volume of PBS. When gradient separation for peripheral blood mononuclear cells (PBMC) was performed prior to CTC enrichment, Ficoll-Paque Premium (GE Healthcare, IL, USA) and SepMate PBMC Isolation tubes (Stemcell Technologies, BC, Canada) were used according to the manufacturer’s recommendations. After gradient separation, PBMC layer was processed according to Celsee recommendations. CTCs were captured in the microfluidic chip and then washed back out of the chip capillaries by reversing the flow of buffer. This washed-back fluid was collected and transferred to a 15 mL tube and spun at 300 g for 5 min. Supernatant was removed and the CTC-enriched sample was kept at −80 °C in a 1.5 mL Eppendorf tube after adding 350 µL of lysis buffer RLT (Qiagen, Netherlands) with 1% β-mercaptoethanol until RNA extraction.

In some experiments, ScreenCell MB kit CTC enrichment was performed, instead of Celsee enrichment, according to the manufacturer’s protocol. Briefly, after removing and replacing the plasma as described above, up to 6 mL of blood was mixed with PBS if needed to achieve a final total volume of 6 mL. This sample was then mixed with 1 mL of ScreenCell LC dilution buffer. The samples were homogenized by inverting the tube 5 times and incubating for 2 min at room temperature. Then the 7 mL of diluted blood with buffer was processed through the ScreenCell filter to capture CTCs. The filter, with captured CTCs, was then transferred in a 1.5 mL conical tube. One hundred microliters of lysis buffer RLT (Qiagen) with 1% β-mercaptoethanol was added to the filter and then the tube was vortexed for 3 sec. Next, the tube was centrifuged at 12000 × *g* for 1 min. One hundred microliter of lysis buffer was added a second time following the same process and then the sample was stored at −80 °C until RNA extraction.

### DNA and RNA extraction

RNA was extracted from cell lines using the RNeasy Mini Kit (Qiagen). RNA from tumor samples were extracted from formalin-fixed paraffin-embedded (FFPE) using the RNeasy FFPE Kit (Qiagen). For the CTC enriched samples, RNA was extracted with the RNeasy Micro Kit (Qiagen). RNA quality was evaluated by automated electrophoresis with High Sensitivity RNA ScreenTape (Agilent, CA, USA). RNA integrity number (RIN) and DV200 were calculated using the TapeStation Analysis Software (Agilent)^7,8^. For sorted single cells, cells were sorted using the SONY SH800S with at least 16 single cells sorted twice in independent experiments. The SingleShot Cell Lysis Kit (Bio-Rad) was used according to the recommendations using 4 µl per cell of lysis buffer containing proteinase K and DNase. The entirety of the RNA product was used for cDNA generation and pre-amplification.

### Digital droplet PCR and pre-amplification

**Fig. 1:**
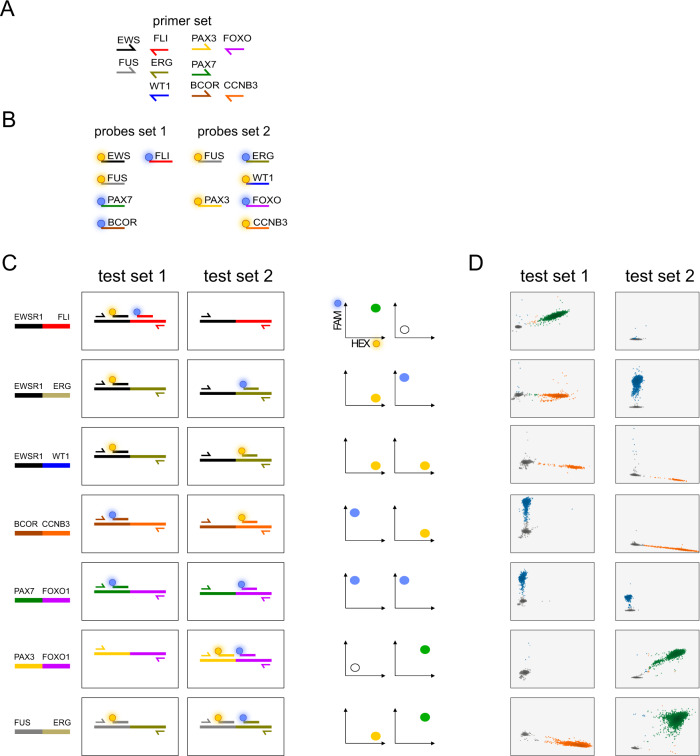
Multiplex droplet digital PCR design and validation. **A** Primers and (**B**) probes were designed to anneal to exons of target genes commonly involved in somatic translocations, including EWSR1, FLI1, ERG, FUS, WT1, PAX3, PAX7, FOXO1, BCOR, CCNB3. **C** The multiplexed ddPCR amplifications are split into two independent reactions labeled PCR1 and PCR2. Each reaction uses the same complete primer set but only a specific subset of probes such that the two PCR reactions produce a unique combination of fluorescent signals (characterized by either FAM, HEX, or both) that is characteristic of a unique set of gene partners. **D** The unique fluorescent signal combination (across PCR1 and PCR2) for each targeted fusion was validated with cells lines or a synthetic DNA construct.

PCR primers and probes were designed with Primer3Plus (http://www.bioinformatics.nl/cgibin/primer3plus/primer3plus.cgi) to amplify disease specific fusion transcripts using Bio-Rad settings for the QX200 Droplet Digital PCR system (Bio-Rad). Primers and probes were designed to target exons that are recurrently involved in the majority of the targeted translocations, with 2 probes per fusion transcript, one designed for each gene (Fig. 1A–C and supplemental Table 1). cDNA was made using SuperScript IV VILO Master Mix (Invitrogen, MA, USA) using the entire volume of RNA extracted for a final volume of 20 µL. For the single cell experiment only, 1 µl of the Master Mix was combined with the 4 µL of RNA extracted. For cell lines, cDNA was made from 500 ng of extracted RNA. For FFPE tissue from unstained slides, cDNA was made using half of the extracted RNA from 2 unstained slides to a maximum of 5 µg. For the experiment using 1/20th of the extracted RNA, 1/10^th^ of the RNA used for the first RT-PCR, corresponding to 1/20^th^ of the RNA from 2 to 5 unstained slides was used.

Pre-amplification of cDNA was performed using TaqMan PreAmp Master Mix (Applied Biosystems) with an adapted protocol. Ten microliters of 2X Master Mix was combined with 1 µL of mixed multiplex primers at 45 nM each and 9 µL of cDNA product. For the single cell experiments, the entirety of the 5 µL of cDNA was used. Thermal cycling conditions were: 10 min at 95 °C, 14 cycles of 15 s at 95 °C and 4 min at 60 °C, then 10 min at 99 °C and hold at 4 °C. Pre-amplified product was then diluted at 1:20 in TE. ddPCR for the multiplex assay was performed using 2 µL of template, either diluted preamplifed material or cDNA for non pre-amplified, with the ddPCR Supermix for Probes (No dUTP) (Bio-Rad) and the QX200 Droplet Digital PCR system (Bio-Rad). PCR-based experiments, including the use of this pre-amplification step, are expected to increase the risk of experimental contamination. Efforts within the lab were used to prevent these issues, including dedicating separate workspaces for pre- and post-amplification steps of the experiments. To further address this concern, multiple negative and positive control wells were included on every ddPCR run, allowing for the identification of contaminated experiments. Entire runs were excluded from further interpretation if unexpected ddPCR signals were detected in positive and/or negative control wells.

EWSR1-FLI1-specific ddPCR was performed using ddPCR™ EvaGreen Supermix (Bio-Rad) and the QX200 Droplet Digital PCR system (Bio-Rad). For mouse CTC experiment, 8 µL of cDNA made from all extracted RNA was used for the EWSR1-FLI1 specific ddPCR and 5 µL was used of the multiplex ddPCR, exhausting the material. Primers were used at a final concentration of 900 nM and the concentration for each probe was 500 nM. Cycling conditions were: 10 min at 95 °C, 36 cycles of 30 sec at 95 °C and 1 min 30 s at 60 °C, then 10 min at 98 °C and hold at 4 °C. Analysis was performed using the QuantaSoft program (Bio-Rad).

## Results

### Multiplex droplet digital PCR assay for translocation detection

To create an assay that met our target specifications, we decided to develop a workflow that utilized dual color ddPCR, a technology now frequently available in clinical molecular pathology laboratories. In this system, PCR primers are designed to amplify regions of interest and probes can be labeled with two colors, either FAM (Fluorescein amidites) or HEX (hexachlorofluorescein). To test the potential for this system to detect and correctly identify fusions and their specific partner genes, we designed a set of primers that would amplify seven fusions that are commonly identified in pediatric solid tumors (Table 1). Primers for all the targeted fusions are present in each reaction such that cDNA containing transcripts of any of these somatic fusions will be amplified. Primers were designed so that amplification of these targeted fusions would occur even though some translocation breakpoints are known to involve multiple exons, avoiding break-point specific primer sequences. We then developed two pools of probes that would be applied in two separate reactions for each sample undergoing testing. Each pool of probes has multiple probe sequences targeting the fusion partner genes, labeled with either FAM or HEX. These pools were created in such a way that the specific combination of signals (FAM, HEX or both) from each of the two pools could only be produced from a single pair of fusion partner genes (Fig. 1A–C). In theory, this approach could be applied to a panel of up to 11 targeted fusion transcripts.

**Table 1:** Targeted fusion transcripts.

Partner gene A	Partner gene B	Diagnosis
EWSR1	FLI1	EWS
EWSR1	ERG	EWS
FUS	ERG	EWS
EWSR1	WT1	DSRCT
PAX3	FOXO1	aRMS
PAX7	FOXO1	aRMS
BCOR	CCNB3	BCOR-CCNB3 sarcoma

Partner gene pairs targeted by multiplexed PCR primers and the corresponding clinical diagnosis associated with each fusion.

*EWS* Ewing Sarcoma, *DSRCT* desmoplastic small round cell tumor, *aRMS* alveolar rhabdomyosarcoma.

In ddPCR, reactions are allocated to individual droplets. Therefore, in this assay, only droplets that contain a fusion for which we have included complementary primers will be amplified (Fig. 1A). Amplified droplets will fluoresce with a specific spectrum (FAM, HEX or both) based on the specific fusion amplified and probe set being tested (Fig. 1B, C), resulting in highly specific fusion partner identification. Furthermore, because the full primer set is run for every sample, each positive control also acts as a negative control for the other fusions in the assay design (for example, an EWS-FLI positive control sample will also be negative for the fluorescent pattern associated with PAX3-FOXO1 and the rest of the targeted partner genes). With this approach, ddPCR is also expected to be extremely sensitive because fusions can be accurately detected and identified even if only a few droplets contain a targeted fusion.

To validate the primers and probes for this assay, cDNA was generated from RNA extracted from translocation-positive cell lines or from synthesized DNA fragments when lines were not available. In all cases, the expected pattern of probe signals matched experimental results and there were no cases of false positives, confirming the specificity of the assay for the targeted fusions (Fig. 1D).

### Multiplexed ddPCR has limited sensitivity for detecting fusions in low tumor content samples

**Fig. 2:**
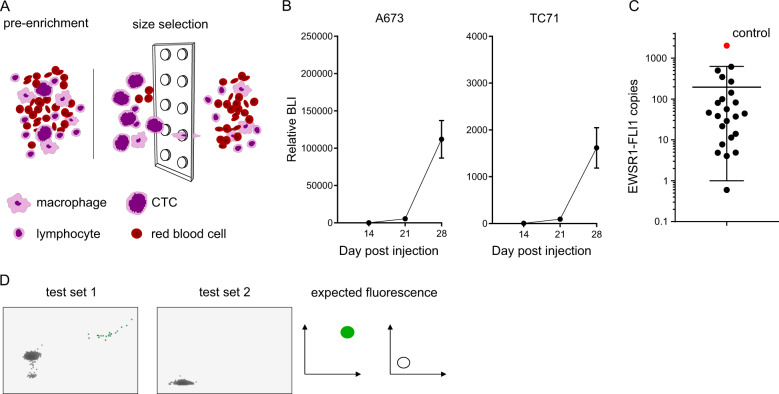
Human xenografted CTCs can be detected in mouse blood by identification of the expected fusion transcript using size selection and ddPCR. **A** Cartoon of the size enrichment of circulating tumor cells using either the Celsee chip or ScreenCell filter. Large tumor cells are not able pass through the 6 µm pores present in either the microfluidic chip or filter. **B** Relative bioluminescence (mean and standard error of the mean) of 23 mice at day 14, 21, and 28 after cell line IV injection of A673 (left panel) and TC71 (right panel) showing very high tumor burden at day 28 in Ewing sarcoma xenograft mouse models. **C** Blood samples collected from mice bearing high burden of metastatic xenografted Ewing sarcoma cells were size enriched and subjected to EWSR1-FLI1-specific ddPCR. Absolute expressions of EWSR1-FLI1 transcript is represented, showing that all 23 samples had detectable CTCs. **D** Multiplex transcript detection by ddPCR. The EWSR1-FLI1 fusion was also detected in four of these same blood samples using the multiplexed ddPCR primer and probe set described in Fig. 1.

We next set out to determine the utility of our approach to detect fusions in samples containing minimal tumor content. We used two sample types, liquid biopsy samples collected from xenograft mouse models of human cancer cell lines and small volumes of archival FFPE tissue. The detection of CTCs in pediatric sarcomas has been previously described using a size and deformability exclusion-based technology^9^. To test whether our assay could detect the presence of CTCs, we utilized an established xenograft model of Ewing sarcoma that yields a high burden of metastatic disease. Blood was collected by cardiac puncture from mice 28 days after tail-vein injection of A673 or TC71 Ewing sarcoma cell lines. CTCs were enriched by microfluidic capture on a commercially available system (Fig. 2A). Day 28 was chosen to maximize the chances to detect CTCs in mice bearing a very high burden of disease, as demonstrated by bioluminescence imaging (Fig. 2B). The EWSR1-FLI1 fusion transcript was detected by ddPCR using EWSR1-FLI1-specific primers in all the samples (Fig. 2C). We then applied multiplexed ddPCR to four of those CTC-enriched blood samples. The EWSR1-FLI1 fusion transcript was identified by our multiplex assay in all 4 cases (Fig. 2D). While this demonstrates the feasibility of using this assay as a non-invasive diagnostic tool, our objective is to detect the presence of a fusion transcript even when input material has very small amounts of tumor content.

**Fig. 3:**
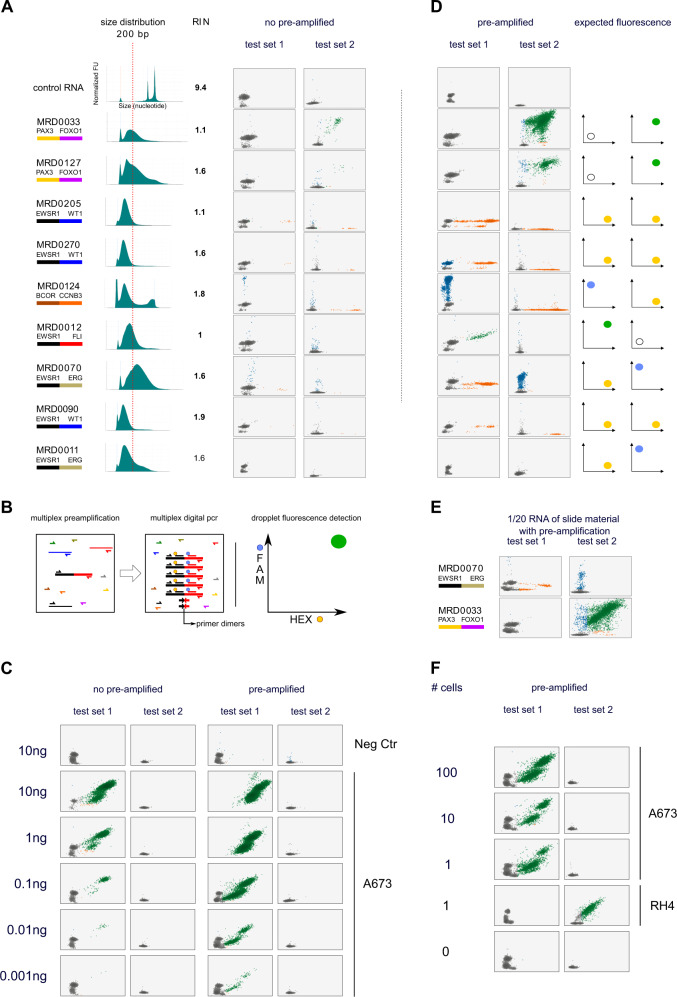
Tiered multiplexed ddPCR improves detection of targeted translocations. **A** Multiplex digital PCR of FFPE samples from patients with translocation positive sarcoma. Left is the known translocation partner with genes color-coded as in Fig. 1C. The middle of the panel shows the size distribution of the extracted RNA by capillary gel electrophoresis with a vertical dotted line indicating 200 base pairs as a reference and the RNA integrity numbers (RIN) indicated to the right of each histogram^7^. On the right of this panel is shown the ddPCR fluorescent measurements for samples after multiplexed amplification with primer-probe sets for PCR1 and PCR2 reactions. **B** Schema depicting the addition of pre-amplification using the same primers as used for ddPCR (but without probes) to first amplify cDNA made from targeted fusion partners prior to analysis with ddPCR. **C** ddPCR results for cDNA made from RNA extracted from a negative control sample (fusion negative cell line, 293T) and the A673 cell line expressing an EWSR1-FLI1 fusion transcript. RNA was serially diluted, prior to making cDNA, to the indicated targeted amount. ddPCR was performed without (left) and with (right) pre-amplification. **D** Pre-amplification was then applied to the same FFPE samples as in panel A, demonstrating increased ddPCR fluorescence signaling matching the expected pattern for each known fusion type in 8 of 11 samples tested. The three samples that remained negative with pre-amplification are depicted in Fig. 4C.**E** Examples of translocations detected in samples for which cDNA was made from 1/20^th^ of the original extracted RNA. **F** Tiered multiplexed ddPCR performed on A673 and RH4 cells flow sorted into wells with the indicated number of cells. The expected transcript was detected at a single cell level.

We next tested the ability of multiplexed ddPCR to detect cancer-specific transcripts from minimal volumes of archival tissue. We collected two to five unstained slides from nine pre-treatment and two post-treatment biopsies from patients with EWS, fusion-positive RMS, DSRCT and BCOR-CCNB3 sarcoma. Slides had been created one to six years ago (mean 3.8 years) and had been stored at room temperature. As expected, RNA from these FFPE tissues were highly degraded with a mean RNA Integrity Number (RIN) of 1.58 (range 1 to 2.7), as compared with 9.4 for control RNA from fusion negative cell line 293T, and a mean DV200 index (percentage of RNA fragments >200 nucleotides in size) of 34% (range 6–75; Fig. 3A)^7,8^. Three samples had a DV200 below 10% whereas control RNA was at 98%. We performed multiplexed ddPCR for the targeted fusions using up to half of the RNA extracted from the available slides (maximum input of 5 µg of RNA). Although many of the samples demonstrated evidence of the expected fluorescence pattern for the known translocation, the number of positive droplets was too low in the majority of cases to confidently identify the correct fusion type (Fig. 3A and Table 2). These results suggest that our current version of the assay would have limited sensitivity for fusion detection in samples with extremely low tumor content or degraded nucleic acid.

**Table 2:** FFPE tumor tissue characteristics.

Sample	Clinical scenario	Pathology data	RNA conc	RIN	DV_200_	No pre-amplification	Pre-amplification	Final result^a^
MRD0033	Resection at diagnosis	PAX3-FOXO1	322	1.1	52.46	PAX3-FOXO1	PAX3-FOXO1	PAX3-FOXO1
MRD0127	Resection after chemotherapy	PAX3-FOXO1	86.4	1.6	56.13	PAX3-FOXO1	PAX3-FOXO1	PAX3-FOXO1
MRD0205	Diagnostic biopsy	EWSR1-unknown	9.32	1.1	8.25	EWSR1-WT1	EWSR1-WT1	EWSR1-WT1
MRD0270	Diagnostic biopsy	EWSR1-WT1	5.92	1.6	6.46	EWSR1-WT1	EWSR1-WT1	EWSR1-WT1
MRD0124	Biopsy of a third recurrence	BCOR-CCNB3	too low	1.8	37.04	BCOR-CCNB3	BCOR-CCNB3	BCOR-CCNB3
MRD0012	Metastasectomy at progression	EWSR1-FLI1	472	1	29.44	0	EWSR1-FLI1	EWSR1-FLI1
MRD0070	Resection at diagnosis	EWSR1-ERG	752	1.6	75.45	0	EWSR1-ERG	EWSR1-ERG
MRD0090	Diagnostic biopsy	EWSR1-WT1	too low	1.9	9.08	0	EWSR1-WT1	EWSR1-WT1
MRD009	Resection after chemotherapy	EWSR1-FLI1	492	1.2	31.4	0	0	EWSR1-FLI1
MRD004	Resection after chemotherapy	EWSR1-FLI1	14.6	2.7	29.59	0	0	EWSR1-FLI1
MRD0011	Diagnostic biopsy	EWSR1-ERG	13.5	1.8	31.47	0	0	0

Clinical information, quality metrics describing the extracted RNA from each sample, and the multiplexed ddPCR results for each FFPE tumor biopsy sample profiled in this study.

^a^Final results include the use of a second set of primers designed to target a short template for EWSR1-FLI1 and EWSR1-ERG.

RNA conc: RNA concentration; RIN: RNA integrity number; DV_200_: percentage of RNA fragments >200 nucleotides in size.

### Tiered multiplexed amplification improves sensitivity for fusion partner detection by ddPCR

We hypothesized that the addition of fusion-specific multiplexed PCR amplification (pre-amplification) performed prior to ddPCR (tiered multiplexed amplification) would improve the sensitivity of our assay for fusion detection in samples with extremely low tumor content without compromising specificity (Fig. 3B)^10^. One additional advantage of this approach is that pre-amplification could be performed with a larger number of PCR partners because this step is not constrained by the number of available fluorescent signals detected by ddPCR. If successful, this step could be used to target the amplification of up to one hundred candidate somatic fusions, creating an amplified sample that could be tested for the presence of a cancer-specific fusion across multiple panels of primer/probe ddPCR assays.

We first tested the addition of the pre-amplification step in serially diluted RNA extracted from two fusion positive cell lines, A673 (EWSR1-FLI1) and RH4 (PAX3-FOXO1), ranging from 10 ng to 1 pg. We found that without amplification, fusion detection was possible with 0.01 ng but with pre-amplification, fusions were detectable with only 1 pg of extracted RNA, less than the total expected RNA content of a single cell (Fig. 3C)^11,12^.

Next, we repeated the detection of fusions from RNA extracted from FFPE tissue with our tiered amplification approach. Using this method, we were able to detect the expected oncogenic fusion in 8 of 11 fusion-positive samples, demonstrating an improvement in the sensitivity of our assay and confirming the ability to detect the EWSR1-FLI1, EWSR1-ERG, EWSR1-WT1, PAX3-FOXO1 and BCOR-CCNB3 fusions from patient samples despite the poor quality of the RNA (Fig. 3D). To further challenge the sensitivity of the workflow, we repeated the experiment using only 1/20th of the total extracted RNA from two of these samples and were still able to detect the expected translocations (Fig. 3E). Finally, we collected a decreasing number of A673 and RH4 cancer cells by flow sorting, extracted the RNA for each sample, and performed tiered multiplexed analysis. We found that we could accurately detect the presence of the EWSR1-FLI1 fusion or PAX3-FOXO1 transcript from the RNA extracted from a single cell, respectively 26/32 positive for A673 and 47/48 for RH4 (Fig. 3F).

### Tiered multiplexed ddPCR fusion detection is adaptable to redesign

**Fig. 4:**
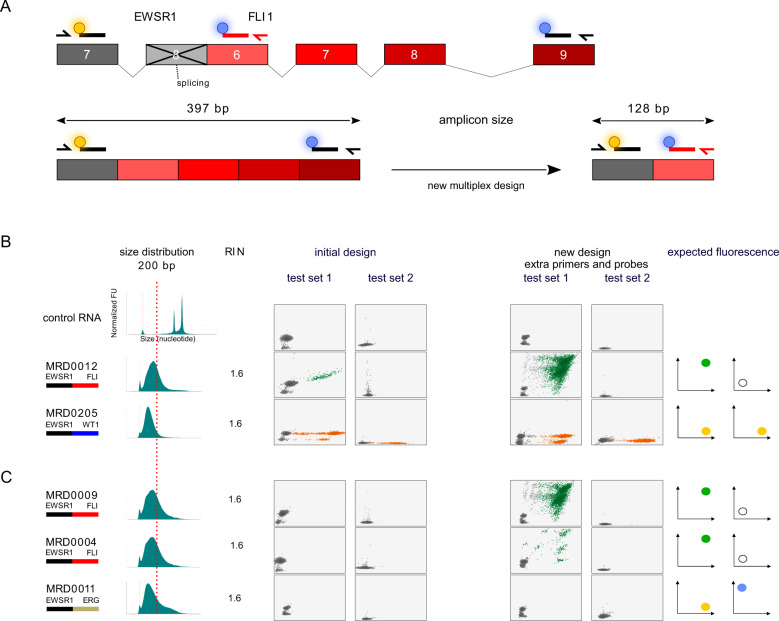
Tiered multiplexed ddPCR can be redesigned to improve detection of specific fusion partner breakpoints. **A** Cartoon showing the rationale for designing an additional FLI1-targeting primer and probe. The initial design is indicated by black colored primers and probes and the new design is indicated by a red primer and probe. The new design results in partner primers and probes binding less than 130 base pairs from each other when the targeted fusion transcript breakpoint is between exon 7 of *EWSR1* and exon 6 of *FLI1*. In this way, complementary primers and probes can successfully bind to cDNA made from RNA strands with a fragment length of 200 base pairs or less to accommodate fusion transcript. **B** The addition of the new FLI1-targeting primer and probe to the initial tiered multiplexed primer and probe pools does not disrupt detection of the expected fusion in samples that were previously positive and (**C**) improves the fusion detection for two EWSR1-FLI1 positive tumor biopsy samples as intended. It was still not possible to detect the EWSR1-ERG fusion transcript for MRD0011 sample despite a reduction of the amplicon size from 281 bases to 187 bases.

We hypothesized that one of the reasons that fusions remained undetectable in 3 of 11 samples, even after the addition of pre-amplification, was that the fragment length of the RNA extracted from these samples was significantly shorter than the expected length needed for both primers to bind to each end of a fusion-positive cDNA strand. Indeed, we found that the DNA breakpoints implicated in the two EWSR1-FLI1 positive cases and one EWSR1-ERG, included exons that would separate the PCR primer pairs and probes of the targeted fusion transcript wider than the base pair length of the measured RNA fragments extracted from the samples (Fig. 4A). To address this problem, we redesigned the FLI1 and ERG primers and probes to hybridize to an exon closer to the breakpoint for the most common EWSR1-FLI1 and EWSR1-ERG fusion type resulting in an expected amplicon size more likely to be compatible with fragmented RNA (Fig. 4A). These primers and probes were added to our existing tiered multiplexed assay without removing the previous EWSR1, FLI1 and ERG targeting primers or probes. First, we tested these new master mixes on two samples for which we were previously able to detect EWSR1-fusions using the original design to make sure the new design did not disrupt the functioning of our assay. We found that the new design did not interfere with the detection of an EWSR1-FLI1 fusion or an EWSR1-WT1 fusion (Fig. 4B). Next, we tested the design on RNA extracted from tumor samples for which the expected fusions had not previously been detected. The new assay design was able to detect the EWSR1-FLI1 fusion partners in the two EWSR1-FLI1 positive samples demonstrating the ease with which this assay can be adapted as new fusion partners or fusion breakpoints are identified (Fig. 4C). However, for the sample with a known EWSR1-ERG fusion (MRD0011), we were not able to detect the fusion transcript with this new design despite a reduction of the amplicon size from 281 bases to 187 bases.

### Tiered multiplexed ddPCR can detect pathognomonic fusions from liquid biopsy samples of patients with fusion positive sarcomas

**Fig. 5:**
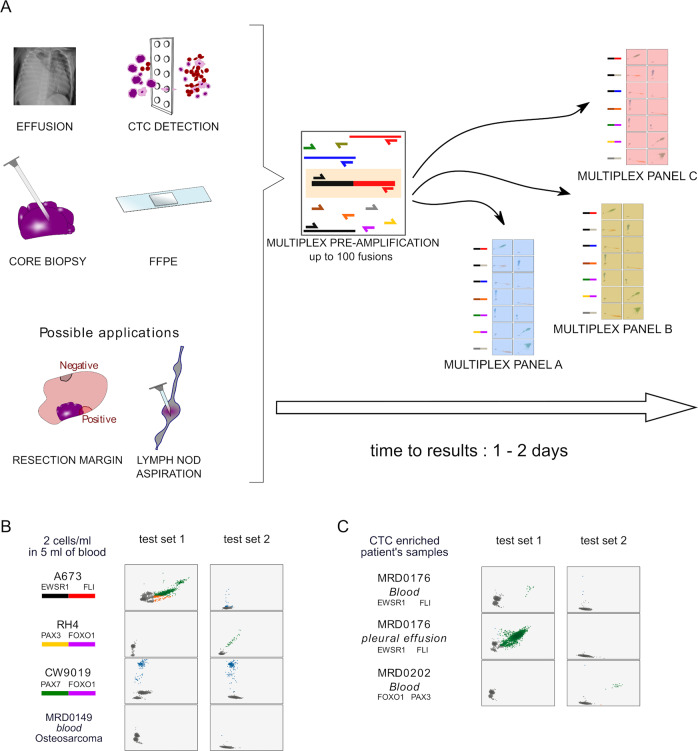
Tiered multiplexed ddPCR can detect disease-defining somatic fusions from multiple tissue types. **A** Schematic depicting (left) the theoretical applications for which tiered multiplex ddPCR could be applied in order to detect cancer cells harboring pathognomonic somatic fusions. Although ddPCR probe and panel sets are restricted to the detection of 11 targeted fusion partner genes (far right of this panel), the pre-amplification step could be developed with primers targeting up to one hundred fusion partners (middle), creating an amplicon reservoir that could be tested with multiple ddPCR primer and probe sets. **B** ddPCR plots demonstrating the expected fluorescence pattern for each expected translocation detected from donor blood samples spiked with the indicated cancer cell line at a concentration of 2 cells per mL of blood. As a control, the experiment was also conducted with a blood sample from a patient treated for osteosarcoma that does not express a fusion targeted by our primer and probe ddPCR panel. **C** Tiered multiplexed ddPCR detects evidence CTCs from liquid biopsy samples collected from patients with EWS and fusion positive RMS.

One potential use of this assay is to identify disease-defining fusions through non-invasive tumor sampling. We hypothesized that our tiered multiplexed PCR assay could detect targeted fusions from CTCs collected in peripheral blood samples and other body fluids in patients with fusion-positive sarcomas (Fig. 5A). To test this, we first collected peripheral blood from a donor without cancer and added cancer cell lines to the sample. A673 (EWSR1-FLI1), CW9019 (PAX7-FOXO1) and RH4 (PAX3-FOXO1) cells were collected and quantified such that 10 cells were added to 5 mL of peripheral blood (2 cells per mL). Samples were first enriched by size selection and then RNA was extracted from the enriched samples for testing in our assay. We detected the presence of the expected translocation in all three samples without false positive (Fig. 5B).

To determine whether translocations could be detected from liquid biopsy samples in patients with known fusion positive sarcomas, we collected peripheral blood and fluid samples from 24 patients with a translocation positive sarcoma from March 2018 to September 2019. We processed 25 blood samples and 3 pleural effusions from these patients. RNA was extracted from the 3 pleural fluid samples without enrichment while the peripheral blood samples were first enriched for potential CTCs by size selection. Of these samples, six peripheral blood samples were drawn at the time of diagnosis and 19 were collected at progression or relapse. At diagnosis, we detected the presence of the expected fusion from 2 out of 6 (33%) peripheral blood samples (Fig. 5C). At relapse, we detected the expected fusions in 4 out of 19 blood samples (21%). We also detected the expected fusions in all the pleural effusions, 2 EWSR1-FLI1 and one BCOR-CCNB3. No false positives were found in any sample. The time to result after sample collection was one to two days.

**Fig. 6:**
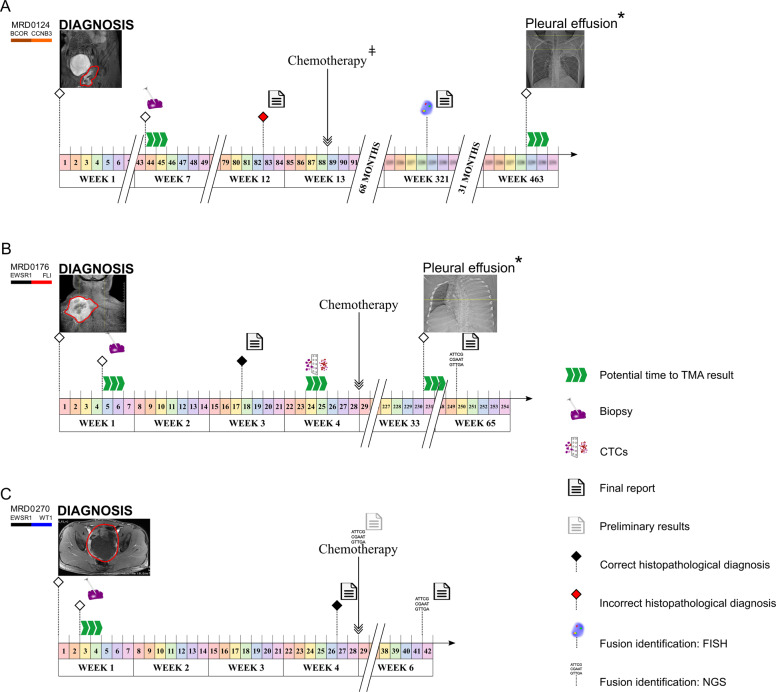
Potential impact of tiered multiplexed ddPCR in the clinical care of patients with fusion-positive sarcomas. Clinical timelines illustrating radiologic, diagnostic, pathologic, and treatment milestones in example patients with fusion-positive pediatric sarcomas. Triple green arrows indicate the predicted time to tiered multiplexed ddPCR results if the assay had been implemented in the clinical pathology lab with return of results. **A** The initial clinical diagnosis and treatment for patient MRD0124 was osteosarcoma. Tiered multiplexed ddPCR from the initial diagnostic procedures was obtained and identified the presence of a BCOR-CCNB3 translocation, contradicting the histologic diagnosis. The diagnosis of a BCOR-CCNB3 sarcoma was later made at relapse (week 321) by FISH. After responding to relapse therapy, the patient then developed pleural effusions that were negative for cancer during cytologic examination but tested positive for the BCOR-CCNB3 fusion by tiered multiplexed ddPCR in cells isolated from the same effusion sample. This was later confirmed to be a site of further disease progression. **B** The diagnosis of Ewing sarcoma for patient MRD0176 was made by histology after 3 weeks and chemotherapy was started without detection of a Ewing-specific fusion. Tiered multiplexed ddPCR identified an EWS-FLI1 fusion from the initial biopsy tissue and from a pre-treatment blood sample containing CTCs. Later, the patient developed pleural effusions that were negative for cancer cells by cytologic examination but tested positive for the EWS-FLI1 fusion by tiered multiplexed ddPCR in cells extracted from the same effusion sample. This was later confirmed to be a site of further disease progression. Clinically, the EWSR1-FLI1 fusion was identified by NGS performed on tumor tissue only after the patient died from disease. **C** The histologic diagnosis of DSRCT for patient MRD0270 was made 4 weeks after initial diagnostic biopsy was performed. Detection of an EWS-WT1 fusion by NGS was preliminary at the time of treatment initiation and later finalized six weeks after biopsy. Tiered multiplexed ddPCR detected the pathognomonic EWS-WT1 fusion in the diagnostic sample. TMA tiered multiplexed ddPCR assay, NGS next-generation sequencing, CTCs circulating tumor cells, DSRCT desmoplastic small round cell tumor. ǂ The chemotherapy used was not standard of care for the patient’s final diagnosis. * Cytology did not find any malignant cells.

While these experiments were largely done retrospectively in a research laboratory environment, we also reviewed clinical and radiological data from these patients to determine whether prompt clinical implementation of this assay could have impacted patient care. In multiple cases, we found that tiered multiplexed ddPCR was able to detect pathognomonic fusions in samples from which existing diagnostic workflows resulted in either a protracted delay to diagnosis or misleading diagnostic information that likely impacted the initiation of therapy (Fig. 6).

## Discussion

In this study, we have developed a novel tiered multiplexed ddPCR approach to identify somatic fusion partner genes from cancer samples with an unknown translocation status. We developed this assay to feature specific characteristics that we believe fill an unmet need among the existing methods typically leveraged to identify somatic fusions in the clinical and research settings. These features include the need for minimal amounts of cancer content per sample, a high performance for samples with a low quality of extracted nucleic acids, results available within two days of receiving the tissue sample, no specific tissue processing or handling requirements, no need for knowledge of patient-specific fusion breakpoints, flexibility of the assay to be frequently redesigned to detect new fusion partners, and a high specificity without the requirement of an advanced computational pipeline. We believe that such an assay could complement the repertoire of molecular tools available to pathologists working to make a diagnosis for a tumor for which there is suspicion for a pathognomonic translocation. Furthermore, accurate detection of translocation partners can also aid in the selection of treatment, subclassification within a cancer diagnosis, and in prognostication^5,13–24^.

As the number of distinct sets of recurrent somatic gene fusions grows, the need to detect the presence of both fusion partners for a given cancer has become increasingly important to making an accurate diagnosis. For example, during the work-up of a small round blue cell cancer, for which Ewing sarcoma may be one of the possible diagnoses, pathologists frequently interrogate biopsy samples with *EWSR1*-targeting FISH probes that “break apart” in the presence of an *EWSR1*-translocation^20,25,26^. However, without further testing, this result is not diagnostically conclusive because the *EWSR1* gene is known to have multiple fusion partners depending on the cancer type. Ewing sarcoma is defined by fusions of *EWSR1* with either *FLI1* or *ERG* while DSRCT is defined by fusions of *EWSR1* and *WT1*. One of the strengths of our tiered multiplexed digital PCR approach is that the pattern of the probe signal detected is unique to the amplification of a translocation between specific partner genes. While the two-probe digital PCR design limits each primer-probe set to 11 specific translocation partners, the pre-amplification step could be multiplexed to create an amplified product for up to 100 fusion partner pairs. This amplified material could then be analyzed by multiple primer-probe sets, either in parallel or in series, depending on the urgency for obtaining results. Primer-probe sets for the ddPCR step of our assay could be designed to simultaneously test for fusions present in tumors that commonly present with similar clinical features. In this way, the most pertinent panel or panels could be selected for use in a new diagnostic case based on the presenting history and imaging characteristics. Furthermore, our own effort to improve our primer-probe design for detecting degraded EWSR1-FLI1 transcripts demonstrates the ease with which this assay can be updated with additional fusions of interest when needed.

Another strength of our approach is that tiered multiplexed PCR allows us to detect fusion partners from samples with very little tumor content. We found that we could accurately detect these translocations in RNA samples quantified to be less than the expected content of a single cell. We also found that we could optimize the detection of fusions even in samples with degraded RNA as might be expected in older FFPE tissue or liquid biopsy samples that are not processed immediately after collection. We also describe clinical cases from our institution for which this assay could have facilitated earlier diagnoses, earlier initiation of therapy, more accurate therapy choices, or the detection of early relapse, demonstrating the potential for this assay to improve clinical care for patients with fusion positive cancers. Because our assay requires negligible tissue input, we also believe it could be run in parallel with histologic staining without the fear of consuming tissue that may otherwise be needed for other diagnostic assays, especially when small core needle biopsies were used to obtain diagnostic material. This could further accelerate the time to diagnosis and initiation of treatment for patients with fusion positive cancers.

We also found that we could identify disease-defining fusions in over 20% of blood samples collected from patients with newly diagnosed disease or prior to the initiation of relapse therapy. Although the frequency with which CTCs are present in the blood of newly diagnosed patients is still unknown in most malignancies, our experiments suggests that this assay could allow for non-invasive identification of pathognomonic fusions, prior to attempting a surgical biopsy, in a subset of patients with a suspected diagnosis of a fusion-positive cancer^27–32^.

There are also some expected limitations of this novel approach to detection of characteristic somatic fusions. One limitation is that we can only identify known fusion partners. Other technologies, such as anchored multiplex PCR–based targeted next-generation sequencing, can detect fusions with amplifications anchored by primers targeting only one of the fusion partner genes, allowing detection of novel gene partners^33^. However, this approach requires next-generation sequencing of the amplicon which increases cost, prolongs the time to obtaining a result, and requires significant computational and molecular expertise to analyze and interpret the results. It is also reported to need larger amounts of high-quality RNA than our assay and may also be less adaptable to the addition of new anchor gene targets^34^. Thus, we believe that our tiered multiplex assay sacrifices the potential for discovering new fusion partners in exchange for features that may positively impact patient care. Another limitation to the clinical or commercial implementation of this assay is the need for stringent quality control metrics and sample handling techniques to minimize the risk of contaminations from multiple rounds of PCR amplification. However, we have found that appropriate use of negative and positive controls and implementation of such preventative measures has successfully mitigated this issue in our research laboratory setting. Moreover, if new target fusions need to be added to the assay design, standardized internal controls can easily be designed for each of these new fusions. Finally, to further minimize the risk of sample contamination, we would propose the use of uracil-DNA N-glycosylase treatment and the incorporation of dUTP during the preamplification step in future iterations of this assay, which would assure the rapid detection of PCR contaminations^35^.

In summary, we have developed a novel assay for the detection of somatic fusion partners in tissue samples with low tumor content and low-quality nucleic acids that can be performed at a relatively low cost in two days or less. We believe that this assay may provide pathologists and clinicians with an additional tool for making a diagnosis in a patient suspected of having a fusion positive cancer, assist with prognostication and treatment selection, and could be adapted to less resourced settings where the costs and computational expertise needed for next-generation sequencing are currently prohibitive.

## Supplementary information

Supplementary Table 1: Primer and probe sequences.

## Data Availability

All data generated or analyzed during this study are included in this published article.

## References

[1] Delattre O (1992). Gene fusion with an ETS DNA-binding domain caused by chromosome translocation in human tumours. Nature.

[2] Galili N (1993). Fusion of a fork head domain gene to PAX3 in the solid tumour alveolar rhabdomyosarcoma. Nat Genet.

[3] Machado I, Navarro S, Llombart-Bosch A (2016). Ewing sarcoma and the new emerging Ewing-like sarcomas: (CIC and BCOR-rearranged-sarcomas). A systematic review. Histol Histopathol.

[4] Pierron G (2012). A new subtype of bone sarcoma defined by BCOR-CCNB3 gene fusion. Nat. Genet..

[5] Williamson D (2010). Fusion gene-negative alveolar rhabdomyosarcoma is clinically and molecularly indistinguishable from embryonal rhabdomyosarcoma. J. Clin. Oncol. J. Am. Soc. Clin. Oncol..

[6] Jo VY, Doyle LA (2016). Refinements in sarcoma classification in the current 2013 world health organization classification of tumours of soft tissue and bone. Surg. Oncol. Clin. N. Am..

[7] Schroeder A (2006). The RIN: an RNA integrity number for assigning integrity values to RNA measurements. BMC Mol Biol.

[8] Matsubara, T. et al. DV200 Index for Assessing RNA Integrity in Next-Generation Sequencing. *BioMed Res. Int*. **2020**, 9349132 (2020). 10.1155/2020/9349132 PMC7063185 32185225

[9] Hayashi M (2017). Size-based detection of sarcoma circulating tumor cells and cell clusters. Oncotarget.

[10] Andersson D (2015). Properties of targeted preamplification in DNA and cDNA quantification. Expert Rev. Mol. Diagn..

[11] RNA Yields from Tissues and Cells - FR. // www.thermofisher.com/fr/fr/home/references/ambion-tech-support/rna-isolation/general-articles/rna-yields-from-tissues-and-cells.html.

[12] RNeasy Kits. https://www.qiagen.com/us/products/discovery-and-translational-research/dna-rna-purification/rna-purification/total-rna/rneasy-kits/.

[13] Yoshihara K (2015). The landscape and therapeutic relevance of cancer-associated transcript fusions. Oncogene.

[14] Knott MML (2019). Targeting the undruggable: exploiting neomorphic features of fusion oncoproteins in childhood sarcomas for innovative therapies. Cancer Metastasis Rev..

[15] Malouf C, Ottersbach K (2018). Molecular processes involved in B cell acute lymphoblastic leukaemia. Cell Mol. Life Sci..

[16] Gallego S (2018). Fusion status in patients with lymph node-positive (N1) alveolar rhabdomyosarcoma is a powerful predictor of prognosis: Experience of the European Paediatric Soft Tissue Sarcoma Study Group (EpSSG). Cancer.

[17] Kubo T, Shimose S, Fujimori J, Furuta T, Ochi M (2015). Prognostic value of PAX3/7-FOXO1 fusion status in alveolar rhabdomyosarcoma: systematic review and meta-analysis. Crit. Rev. Oncol. Hematol..

[18] Sorensen PHB (2002). PAX3-FKHR and PAX7-FKHR Gene Fusions Are Prognostic Indicators in Alveolar Rhabdomyosarcoma: A Report From the Children’s Oncology Group. J. Clin. Oncol..

[19] Sumegi J (2010). Recurrent t(2;2) and t(2;8) translocations in rhabdomyosarcoma without the canonical PAX-FOXO1 fuse PAX3 to members of the nuclear receptor transcriptional coactivator family. Genes Chromosomes Cancer.

[20] Chen S (2016). Ewing sarcoma with ERG gene rearrangements: A molecular study focusing on the prevalence of FUS-ERG and common pitfalls in detecting EWSR1-ERG fusions by FISH. Genes Chromosomes Cancer.

[21] Antonescu CR (2017). Sarcomas with CIC-rearrangements are a distinct pathologic entity with aggressive outcome: a clinicopathologic and molecular study of 115 cases. Am. J. Surg. Pathol..

[22] Miettinen M (2019). New fusion sarcomas: histopathology and clinical significance of selected entities. Hum Pathol.

[23] Gatalica Z, Xiu J, Swensen J, Vranic S (2019). Molecular characterization of cancers with NTRK gene fusions. Mod. Pathol J. U S Can. Acad. Pathol. Inc.

[24] Yang JJ, Park TS, Wan TSK (2017). Recurrent Cytogenetic Abnormalities in Acute Myeloid Leukemia. Methods Mol Biol Clifton NJ.

[25] DuBois, S. G. et al. Patterns of Translocation Testing in Patients Enrolling to a Cooperative Group Trial for Newly Diagnosed Metastatic Ewing Sarcoma: A Report From the Children’s Oncology Group. *Arch. Pathol. Lab. Med*. 10.5858/arpa.2020-0671-OA (2021). 10.5858/arpa.2020-0671-OA PMC9048754 33769463

[26] Desmaze C (1994). Interphase molecular cytogenetics of Ewing’s sarcoma and peripheral neuroepithelioma t(11;22) with flanking and overlapping cosmid probes. Cancer Genet Cytogenet.

[27] Mihály D, Nagy N, Papp G, Pápai Z, Sápi Z (2018). Release of circulating tumor cells and cell-free nucleic acids is an infrequent event in synovial sarcoma: liquid biopsy analysis of 15 patients diagnosed with synovial sarcoma. Diagn. Pathol.

[28] Dubois SG, Epling CL, Teague J, Matthay KK, Sinclair E (2010). Flow cytometric detection of Ewing sarcoma cells in peripheral blood and bone marrow. Pediatr. Blood Cancer.

[29] Chalopin A (2018). Isolation of circulating tumor cells in a preclinical model of osteosarcoma: Effect of chemotherapy. J. Bone Oncol..

[30] Yaniv I (2004). Tumor cells are present in stem cell harvests of Ewings sarcoma patients and their persistence following transplantation is associated with relapse. Pediatr. Blood Cancer.

[31] Schleiermacher G (2003). Increased risk of systemic relapses associated with bone marrow micrometastasis and circulating tumor cells in localized ewing tumor. J. Clin. Oncol..

[32] Vincenzi B (2012). 1512 - circulating tumor cells in soft tissue sarcomas patients. Ann Oncol.

[33] Lam SW (2018). Molecular analysis of gene fusions in bone and soft tissue tumors by anchored multiplex pcr-based targeted next-generation sequencing. J. Mol. Diagn..

[34] Afrin S (2018). Targeted next-generation sequencing for detecting MLL gene fusions in leukemia. Mol. Cancer Res.

[35] Andersson D, Svec D, Pedersen C, Henriksen JR, Ståhlberg A (2018). Preamplification with dUTP and Cod UNG enables elimination of contaminating amplicons. Int. J. Mol. Sci..

