# Supplementary material for: Rapid and highly sensitive approach for multiplexed somatic fusion detection
Source: Mod Pathol. 2022 Mar 28;35(8):1022–33. doi: 10.1038/s41379-022-01058-y (PMC9314249; doi:10.1038/s41379-022-01058-y)
Supplement: Supplementary file 1 — Supplementary Table 1: Primer and probe sequences. [file 41379_2022_1058_MOESM1_ESM.docx]

**Supplementary Table 1: Primer and probe sequences.**

| Gene | Primer | Probe |
| --- | --- | --- |
| EWSR1 | AAACTGGATCCTACAGCCAAG | AGAGCAGCAGCTACGGGCAG |
| FLI1-exon 9 | CTGGCCACCTCATCGGG | CGCCAACGCCAGCTGTATCAC |
| FLI1-exon 6^†^ | ATGACTCAGTCAGAAGAGGAGCTTGG | ATGACTCAGTCAGAAGAGGAGCTTGG |
| ERG-exon 12 | GATGCAGCTGGAGTTGGAG | CCTCCTGGAGCTCCTGTCGG |
| ERG-exon 10^†^ | TAACTGAGGACGCTGGTCTT | TGCTCAACCATCTCCTTCCACAGT |
| FUS | GGACAGCAGAACCAGTACAA | AGCAGCAGTGGTGGTGGAGG |
| WT1 | GAGCTGGTCTGAACGAGAAA | ACCAGTGTGACTTCAAGGACTGTGA |
| FOXO1 | CTGGATTGAGCATCCACCAA | TGCACACGAATGAACTTGCTGTGT |
| PAX3 | TACAGACAGCTTTGTGCCTC | TGAGGTGAGAGGCCATTGCCAAT |
| PAX7 | CCTCCAACCACATGAACCC | AGGAGACAGGCCGTTGCTGAC |
| BCOR | AAATTCAGACTCTGCTGGGC | CCTCTGTAGAGTGGCTCCACCC |
| CCNB3 | CCATAATGTTTGGTACCACGG | CTGGAAGTCACACCAGTAGTAGCCTC |
| † Second set of primers and probe to target a shorter amplicon  FLI1: NM_002017.5; ERG: NM_001136154.1 | | |
